# Translation, content validity and internal structure of the Brazilian version of the Adolescent Resilience Questionnaire (B-ARQ)

**DOI:** 10.1371/journal.pone.0310450

**Published:** 2025-02-03

**Authors:** Genara Brum Gomes, Luisa Gatti-Reis, Matheus França Perazzo, Marisa Alves Araújo, Flávio Freitas Mattos, Deirdre Gartland, Saul Martins Paiva, Isabela Almeida Pordeus

**Affiliations:** 1 Department of Paediatric Dentistry, Universidade Federal de Minas Gerais, Belo Horizonte, Minas Gerais, Brazil; 2 Department of Dental Public Health, Universidade Federal de Goiás, Goiânia, Goiás, Brazil; 3 Department of Social and Preventive Dentistry, Universidade Federal de Minas Gerais, Belo Horizonte, Minas Gerais, Brazil; 4 Murdoch Children’s Research Institute, Victoria, Parkville, Australia; UFSCar: Universidade Federal de Sao Carlos, BRAZIL

## Abstract

Resilience refers to one’s ability to face life’s challenges and achieve positive outcomes, and has drawn increasing interest from researchers and policymakers. The Adolescent Resilience Questionnaire (ARQ) is a measure that assesses resilience in adolescents according to a multidimensional perspective, encompassing its several different domains. This cross-sectional study aimed to translate and evaluate the measurement properties of the Brazilian version of the ARQ (B-ARQ) for use with Brazilian adolescents. Two native speakers in Brazilian Portuguese language who were also fluent in English language translated the ARQ from English into Portuguese. A committee of experts in validation studies compared the translated versions. A summarized version was produced and back-translated by a translator native of the English language and fluent in Portuguese. The B-ARQ was pre-tested in a sample of 21 adolescents. An expert committee considered the suggestions and defined the final version of the instrument, which was tested in a sample of 210 adolescent students from public and private schools in the city of Dom Pedrito, Brazil. All students filled out the 88-item instrument as well as a socio-demographic questionnaire. Statistical analysis included descriptive statistics of all variables (frequency distribution, floor, ceiling effects), internal consistency, and confirmatory factor analysis of the version with 88 items and the shortened version with 49 items. The short version with 49 items was validated in a cross-sectional study in an adolescent population of high school students using exploratory factor analysis in the Unites States. The 88-item ARQ had poor structural validity with unsatisfactory model fit indices. Therefore, the investigation focused on the short 49-item version of the ARQ (B-ARQ-SV). The final model presented satisfactory RMSEA = 0.042 (p = 0.994, 90% CI: 0.037–0.047) and SRMR of 0.076, despite the low CFI (0.878). The internal consistency was estimated with McDonald’s Omega for each factor: Confidence (ω = 0.480), Negative Cognition (ω = 0.588), Empathy/tolerance (ω = 0.295), Emotional insight (ω = 0.425), Social Skill (ω = 0.235), Family Domain Connectedness (ω = 0.785), Family Domain Availability (ω = 0.847), Peers Domain Connectedness (ω = 0.719), Peers Domain Availability (ω = 0.402), School Domain Supportive environment (ω = 0.677), School Domain Connectedness (ω = 0.013), Community Domain Connectedness (ω = 0.791). One scale showed a ceiling effect (frequency higher than 15.0%), but we identified no critical floor effect. The B-ARQ-SV is a valid (in terms of content and structural validity) and reliable (in terms of internal consistency) measurement instrument to assess resilience in Brazilian adolescents.

## Introduction

The resilience phenomenon has been defined as one’s ability to face life’s challenges and achieve positive outcomes [1, 2]. It refers to coping, adapting, and resisting adversity and the ability to use resources such as family and other support systems to do so [3]. Initial research identified resilience as an individual’s skills and attributes; however, it has since evolved to highlight its dynamic and multidimensional nature [2, 4]. Evidence suggests resilience is a protective factor for mental health in adolescents, with positive outcomes against depression, anxiety, and bullying [5–7]. Recent studies have attempted to better understand protective factors for resilience and to develop strategies to promote resilience in this age group [8, 9]. Because resilience is a psychological attribute that cannot be directly observed and quantified [10], it needs to be measured through instruments such as questionnaires.

The Adolescent Resilience Questionnaire (ARQ) is a self-report instrument originally developed in Australia, designed to assess resilience in adolescents aged between 11–19 years, based in a reflexive model [2]. The instrument was developed based on an ecological-transactional model [2]. The authors carried out an in-depth literature review together with focus groups with individuals representative of the targeted sample. Another strength of the instrument is the consideration of the construct as multidimensional, involving individual and environmental factors [2]. Cronbach’s alpha coefficient was used to assess reliability, ranging from 0.70 to 0.90, except for the Peers/Availability subscale (α = 0.60).

Since its development, the ARQ has been translated into Spanish [11], Persian [12], Romanian [13], Nepali [14], Hindi [4], and Swedish [15]. In addition, previous studies have sought to assess its psychometric properties across the globe [4, 5, 11–16]. In the United States, a short version of the ARQ with 49 items was validated in a cross-sectional study in an adolescent population of high school students (N = 3222) using exploratory factor analysis, presenting adequate measurement properties [16]. For all 12 factors, internal consistency was considered acceptable (α> 0.70–0.80). In addition, the total scores correlated significantly with total correlation coefficient (r>0.63, p<0.001) [16].

Developing a new instrument is time-consuming, requires great effort and dedication, and can be costly [17]. When instruments to assess psychological constructs are at hand, a cross-cultural adaptation and further evaluation of their psychometric properties, such as reliability and validity, is recommended. Multilingual versions of such measures are important in epidemiological research, as they allow results of studies conducted in different cultures throughout the world to be compared, thus enabling better health policy management [10]. Hence, past studies have sought to assess the psychometric properties of the ARQ and its versions across different countries [4, 5, 11–16].

In Brazil, resilience research has involved at-risk adolescent populations in an attempt to better understand its potential as a predictor of better health outcomes [18–20]. In a population of Brazilian children and adolescents’ victims of violence, it is possible that resilience has been established by the quality of support they were offered at home, within their family environment [19]. However, there is a need for a validated instrument to assess resilience in adolescence that encompasses the multiple dimensions of resilience [18] regarding individual and social factors, designed from a defined conceptual model of resilience. This study aimed to translate and evaluate the measurement properties of the Brazilian version of the ARQ (B-ARQ) for use with Brazilian adolescents.

## Materials and methods

The Human Research Ethics Committee of the Universidade Federal de Minas Gerais, Brazil (CAAE-42091414.9.0000.5149) authorized this study, which was carried out according to the principles of the Declaration of Helsinki. A letter was sent to the adolescents and their legal guardians explaining the aim, characteristics, importance, and methods of the study and requesting their participation. Those who agreed to participate provided a written statement of informed consent signed by the legal guardians and a written assent form signed by the adolescent. The recruitment period for participants in this study started on January 6^th^, 2014, and ended on December 30^th^, 2014. This cross-sectional study was reported according to the COSMIN checklist [21]. Regarding eligibility criteria, adolescents aged 12–14 years who were enrolled in public/private schools in the city of Dom Pedrito were eligible to volunteer. Exclusion criteria was the presence of illness, physical limitation or cognitive impairment. Students enrolled in two public and one private school were invited to volunteer.

### Measures

#### Adolescent Resilience Questionnaire (ARQ)

The ARQ was developed based on an ecological-transactional model, considering its construct as multidimensional to measure resilience in adolescents [2]. The ARQ presents 88 items, 5 domains and 12 scales. The ‘individual’ domain assesses the following scales: confidence (8 items), emotional insight (8 items), negative cognition (8 items), social skills (8 items) and empathy/tolerance (8 items). The ‘family’ domain is used to evaluate connectedness (8 items) and availability (3 items). The ‘peers’ domain assesses connectedness (7 items), and availability (8 items) scales. The ‘school’ domain assesses supportive environment (8 items) and connectedness (8 items) scales. The ‘community’ domain assesses connectedness (6 items). Each item has five response options: 1) almost never, 2) not often, 3) sometimes, 4) most of the time, and 5) almost always. The instrument score is the sum of the response options. Therefore, the value ranges from 88 to 440. Higher scores indicate greater resilience [2].

#### Adolescent Resilience Questionnaire short-version

The questionnaire was designed from the 88-item ARQ. It maintains the same five domains where 49 items are distributed as follows: ‘individual’ (22 items), ‘family’ (7 items), ‘peers’ (8 items), ‘school’ (8 items), and ‘community’ (4 items) [16]. Regarding measurement properties, for all 12 factors, internal consistency was considered acceptable (α> 0.70–0.80). In addition, the total scores correlated significantly with total correlation coefficient (r>0.63, p<0.001). Similar to the ARQ-88, each item has five response options: 1) almost never, 2) not often, 3) sometimes, 4) most of the time, and 5) almost always. The instrument score is the sum of the response options. The 88-item and 49-item versions of the instrument in both English and Brazilian Portuguese are available as supplemental material.

### Cross-cultural adaptation

**Fig 1:**
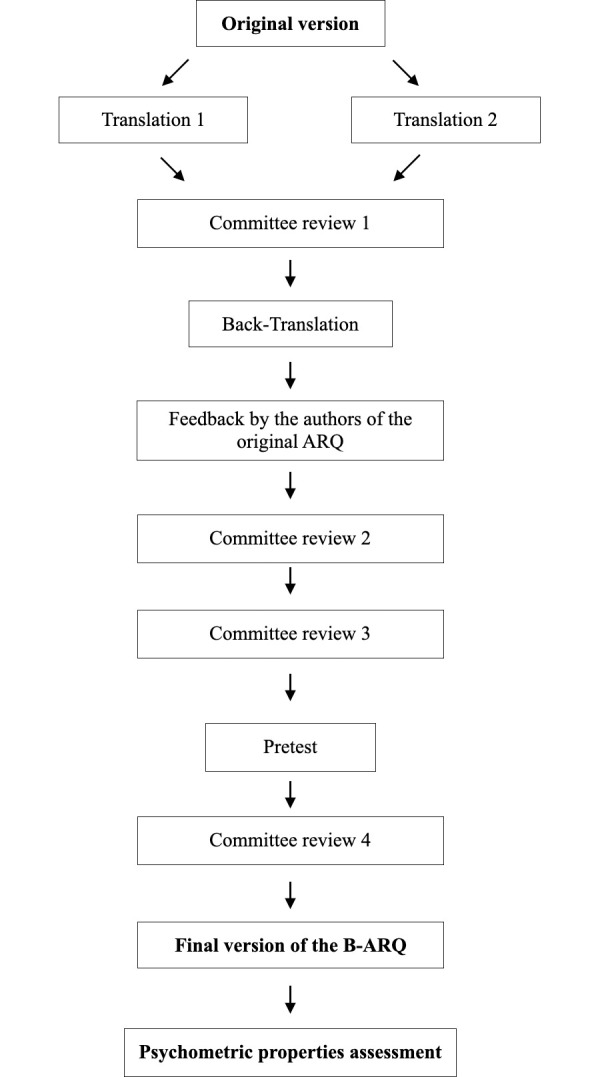
Flowchart of the translation, cross-cultural adaptation and validation of the B-ARQ.

The methods employed for the Brazilian cross-cultural adaptation of the ARQ were based on Beaton et al.’s guideline [22], in addition to the Universalist approach as suggested by Herdman et al. [23], and involved the following steps (Fig 1).

1. Prior to instrument translation, the relevance was assessed and confirmed by an expert in validation studies among adolescents. The translation of the ARQ to Brazilian Portuguese was carried out independently by two native Portuguese-speaking Brazilians fluent in English. The translators were informed regarding the objectives of the instrument and instructed to use language that could be understood by adolescents aged 12 to 14 years. The first translation was carried out by a certified professional translator, and the second by an English teacher with considerable knowledge of semantics—both were fluent in Brazilian-Portuguese and English.

2. A committee composed of two translators, two healthcare professionals with experience in the design/adaptation of health assessment measures, and the leading researcher discussed the translations of the ARQ to develop a summarized version (Committee review 1). Special attention was given to the Brazilian culture and the selection of appropriate words used in the daily life of adolescents.

3. The summarized version was back-translated by a translator native to English with ample knowledge of Brazilian Portuguese (Back-translation). The translator did not know the original ARQ and was not informed regarding the objectives or concepts involved in the instrument.

4. The back-translated version of the ARQ was sent by e-mail to the authors of the original questionnaire for feedback. Then, a committee composed of two healthcare professionals and the leading researcher compared the back-translated version to the original version according to the authors’ feedback (committee review 2). The suggestions were incorporated into the summarized version, which was then assessed by another committee composed of the main researcher, two psychologists, two adolescents, and their respective parents/caregivers (committee review 3). The 88 items were exhaustively discussed for the determination of semantic equivalence. The suggestions of this third committee were discussed among the second committee, who produced the Brazilian version of the ARQ to be administered during the pretest.

5. Comprehensiveness assessment was carried out in the pretest through a cognitive interview, when participants reported how they interpreted each item. The final Brazilian version of the ARQ was administered to a sample of 21 male and female adolescents (pretest). Participants of the pretest were not included in the sample of the main study. The pretest aimed to determine if the instrument was acceptable, including instructions and its items, with appropriate language that could be easily understood by the target sample. At first, adolescents answered the self-administered paper-based instrument. In sequence, each respondent was individually interviewed using a respondent debriefing technique in semi-structured interviews to assess instrument comprehensibility. The interviewer was a dentist experienced in validation studies asked each volunteer regarding the comprehensibility of instrument instructions and items. The comments of the volunteers were transcribed and later analyzed by the same researcher. Respondents reported it took from 15 to 20 minutes to fill out the Brazilian version of the ARQ and did not consider it a problem or a waste of time. However, the adolescents suggested that the instrument could be reduced, considering it was rather long. In addition, volunteers had suggestions related to the response options, and the committee adopted their suggestion: almost never, not often, sometimes, most of the time, and almost always. In Portuguese, the first and second options and the fourth and fifth options are very close in meaning, which could lead to response bias.

6. After the pretest, a committee revised the instrument according to the volunteers’ suggestions (Committee review 4). Thus, the items of the B-ARQ were numbered from 1–88, which did not occur in the original instrument, in addition to the changes in the response options to "very rarely, rarely, sometimes, often, and very often", which led to a more significant distinction and understanding of the options. The changes did not affect the content of the ARQ but rather facilitated the comprehension and administration of the instrument. Thus, a final version of the Brazilian version of the ARQ was obtained and its psychometric properties were tested.

7. The paper-based B-ARQ was self-administered to 210 healthy adolescents in Rio Grande do Sul, Brazil. At school, adolescents received an envelope containing the consent/assent form, in addition to a printed version of the B-ARQ, to be answered at the same time point. When they received the envelope, they were instructed to deliver the consent form to their parents/caregivers. The consent form explained to parents/caregivers the aims of the study and explained that if they allowed it and their child filled out the assent form, the teenager would be able to volunteer in the study. To answer the B-ARQ, adolescents were instructed to not write down their names. In addition, instructions highlighted there were no right/wrong answers, and they should read carefully each question before drawing a circle in the number that best represents the frequency that each item was true to them. An interviewer was not involved at this point. The study included adolescents aged 12 to 14 of either gender from three schools, two private and one public. The choice of this age group was based on more accessible access to adolescents when recruited for future epidemiological studies, as these students are mandatorily enrolled at schools in Brazil. The sample was equally divided among the three age groups (12, 13, and 14 years) to ensure adequate internal distribution (70 individuals of each age group). For validation studies, it has been recommended that sample size should be at least 200 to lower the probability of chance results [24]. Adolescents whose teachers reported having cognitive difficulties that prevented them from answering the questionnaire were excluded from the study. All three schools allowed us to carry out the study through authorization from the principals. Each adolescent answered the paper-based instrument individually. An additional questionnaire addressing socio-demographic factors was self-administered to the volunteers. In case they did not know the answer to a question, such as family income, they could request for assistance of their parents/caregivers.

### Data analysis

The software Statistical Package for the Social Sciences (SPSS for Windows, version 20.0, IBM Corp., Armonk, N.Y, USA) was used for descriptive statistics of all variables (frequency distribution, floor and ceiling effects). The software Mplus (version 8.8) [25] was used for confirmatory factor analysis (CFA) to assess the model fit, using the following indices: Chi-square tests (χ2) for the model, Root Mean Square Error of Approximation (RMSEA), Comparative Fit Index (CFI), and Standardized Root Mean Square Residual (SRMR). We opted to not use exploratory factor analysis since there was a previous study that recommended the model structure with 12 factors and 49 items [16]. According to Hu and Bentler [26], a "relatively good" model fit may be achieved if RMSEA is below 0.06, CFI over 0.95, and SRMR lower than 0.08. Floor and ceiling effects represent how precious instruments are to measure a construct, acceptable floor or ceiling effects are less than or equal to 15% [27]. Reliability was assessed by testing internal consistency, which was evaluated using McDonald’s Omega.

## Results

The majority of the sample was composed of females (53.3%) and individuals who lived in urban areas (96.2%). Mothers were the most common legal guardians who assisted the adolescents to provide socio-demographic details (81.4%). More than half of the parents had 12 years or less of education (57.6%); around half (49.1%) reported a household income ≥ three times the Brazilian monthly minimum wage and more than half of the families had over seven rooms in the home (58.6%) (Table 1).

**Table 1:** Sociodemograph characteristics of the sample (N = 210).

Variables	Frequency (%)	
**Adolescent age**		
12 years	70 (33.3)	
13 years	70 (33.3)	
14 years	70 (33.3)	
**Gender**		
Male	98 (46.7)	
Female	112 (53.3)	
**Housing location**		
Urban	202 (96.2)	
Rural	8 (3.8)	
**Relationship to adolescent**		
Mother	171 (81.4)	
Father	32 (15.2)	
Other	7 (3.4)	
**Parent’s level of schooling**		
≤ 12 years of study	121 (57.6)	
> 12 years of study	89 (42.4)	
Family income (BMW/month)*		
< 3	96 (45.7)	
≥ 3	103 (49.1)	
Does not know/ no answer	11 (5.2)	
**Number of rooms in the house**		
≤ 6 rooms	84 (40.0)	
> 7 rooms	123 (58.6)	
Does not know/ no answer	3 (1.4)	

*BMW Brazilian Minimum Wage, was US$140.00/month at the time of data collection

The 88-item ARQ had a poor structural validity evidence with unsatisfactory model fit index (χ2 = 4474.751 (df = 3588, p < 0.01), CFI = 0.835, RMSEA = 0.034 (p < 0.05, 90%-CI = 0.031–0.038), SRMR = 0.085). Therefore, the focus of the investigation was on the short 49-item version of the ARQ (Table 2). Initially, the first-order model with 12 scales of the 49 ARQ was not positive due to a high correlation (-0.814) between the connectedness and the availability scales in the ‘peers’ domain. We joined both scales considering the theoretical and empirical similarity of the latent variables. The final model presented satisfactory RMSEA (0.042, p < 0.994, 90%-CI = 0.037–0.047) and SRMR (0.076), despite the low CFI (0.878). For reasons regarding the sample size and characteristics of the population explored, items 9, 17, 19, 62 remained on the model even if not significant (p>0.05). The internal consistency was estimated with McDonald’s Omega for each factor: Confidence (ω = 0.480), Negative Cognition (ω = 0.588), Empathy/tolerance (ω = 0.295), Emotional insight (ω = 0.425), Social Skill (ω = 0.235), Family Domain Connectedness (ω = 0.785), Family Domain Availability (ω = 0.847), Peers Domain Connectedness (ω = 0.719), Peers Domain Availability (ω = 0.402), School Domain Supportive environment (ω = 0.677), School Domain Connectedness (ω = 0.013), Community Domain Connectedness (ω = 0.791). Only the scale availability on the ’family’ domain showed a ceiling effect (higher than 15.0%), but no critical floor effects were identified.

**Table 2:** Structural evidence of the B-ARQ-SV based on first-order model with 11 scales.

Domains, scales and items	STD loadings	S.E.	Floor/Ceiling effects (%)	McDonald’s omega (ω)
**Individual Domain**				
Confidence				
7	0.376 (0.217–0.536)	0.081	0.0/0.0	0.480
18	0.497 (0.319–0.674)	0.090		
19	0.148 (-0.022–0.318)	0.087		
22	0.480 (0.315–0.645)	0.084		
39	0.658 (0.493–0.822)	0.084		
Negative Cognition				
20	0.397(0.251–0.544)	0.075	0.5/0.5	0.588
5	0.249(0.074–0.424)	0.089		
8	0.440(0.294–0.587)	0.075		
11	0.569(0.435–0.702)	0.068		
32	0.765(0.599–0.932)	0.085		
Empathy/tolerance				
9	0.103(-0.093–0.268)	0.100	0.0/0.0	0.295
17	0.051(-0.133–0.205)	0.094		
3	-0.697(-0.864–0.556)	0.085		
28	-0.534(-0.673–0.417)	0.071		
Emotional insight				
16	0.554(0.420–0.687)	0.068	0.5/2.4	0.425
10	0.194(0.039–0.349)	0.079		
14	0.305(0.153–0.456)	0.077		
36	0.636(0.494–0.777)	0.072		
Social Skill				
29	-0.328(-0.488- -0.168)	0.082	0.5/0.0	0.235
15	-0.713(-0.842- -0.584)	0.066		
21	0.519(0.381–0.657)	0.070		
33	-0.623(-0.759–0.487)	0.069		
**Family Domain**				
Connectedness				
41	0.741(0.657–0.826)	0.043	0.5/6.2	0.785
42	0.577(0.470–0.684)	0.054		
43	0.771(0.698–0.844)	0.037		
44	0.803(0.729–0.876)	0.037		
Availability				
51	0.937(0.877–0.998)	0.031	1.9/34.3	0.847
47	0.652(0.535–0.769)	0.060		
50	0.911(0.845–0.977)	0.034		
**Peers Domain**				
Connectedness				
52	0.801(0.728–0.873)	0.037	0.5/0.5	0.719
57	0.738(0.640–0.836)	0.050		
61	0.623(0.523–0.724)	0.051		
65	0.670(0.561–0.778)	0.056		
Availability				
56	-0.356(-0.492–0.221)	0.069	1.0/0.5	0.402
58	0.727(0.630–0.823)	0.049		
59	-0.440(-0.571–0.309)	0.067		
62	-0.027(-0.187–0.132)	0.081		
**School Domain**				
Supportive environment				
67	0.741(0.663–0.819)	0.040	0.5/3.8	0.677
71	0.756(0.676–0.837)	0.041		
73	0.546(0.412–0.679)	0.068		
81	0.635(0.533–0.738)	0.052		
Connectedness				
76	-0.788(-0.861–0.715)	0.037	0.0/0.5	0.013
69	0.691(0.606–0.775)	0.043		
74	-0.685(-0.818–0.551)	0.068		
80	0.422(0.291–0.553)	0.067		
**Community Domain**				
Connectedness				
84	0.669(0.578–0.761)	0.047	3.8/2.9	0.791
86	0.861(0.801–0.921)	0.031		
87	0.654(0.564–0.745)	0.046		
88	0.851(0.783–0.920)	0.035		

## Discussion

This is the first study in Brazil to assess the measurement properties–translation, content validity, structural validity, internal consistency, and the factor structure of the ARQ among young people. This study is a paramount step to accumulating structural validity evidence for future studies that use the instrument in this population. The psychometric properties of the Brazilian version of the ARQ and a shortened 49-item version were evaluated following the cross-cultural adaptation of the instrument. The 88-item ARQ presented an unsatisfactory model fit index. However, CFA has shown that the Brazilian version of the ARQ with 49 items (B-ARQ-SV) presented many satisfactory psychometric properties and it is sound for use in a sample of adolescents from Brazil.

First developed in Australia, authors of the original 88-item ARQ assessed its internal consistency through Cronbach’s alpha coefficient, with coefficient values ranging from 0.70 to 0.90, except for the availability scale (α = 0.60) in the ‘peers’ domain [2]. Subsequently, the ARQ in its original structure (88 items) has been validated for use in several countries [11, 12, 14, 15]. However, inconsistencies have been highlighted among researchers [11, 12, 14, 15]. Some studies have confirmed its factor structure but suggested alterations to improve reliability regarding the country’s culture. In the Spanish version, the original factor structure was confirmed [11]. Its internal consistency was overall adequate, with Cronbach’s alpha ranging from 0.60 to 0.84; however, it was insufficient for the empathy/tolerance scale, leading to the suggestion of its revision in the Spanish population. In a similar way, the Nepalese version confirmed the structure with 12 scales with good model fit [14]. However, in its internal consistency analysis, three scales presented α <0.70; which evidenced a need of a revision of items in the confidence scale in the Nepalese version for improved clarity and reliability. In addition, despite confirming the original factor structure of the ARQ, some studies removed items for better performance [12, 15]. In the Iranian version, Cronbach’s alpha was equal to or higher than 0.70 in every scale of the ARQ, except for the ‘community’ domain [12]. The CFA showed that some items of the ’community’ domain had insufficient factor loadings, leading to the exclusion of one item to achieve acceptable Cronbach’s alpha coefficient values. Similarly, the Swedish version found adequate Cronbach’s alpha values for all scales except empathy/tolerance and emotional insights scales [15]. In addition, the study showed an inadequate fit for item 28, and it was excluded to achieve an acceptable model fit.

The unsatisfactory results of the CFA for the 88-item B-ARQ are consistent with previous studies that were not able to confirm the original factor structure of the ARQ [4, 16]. This led studies to suggest shortened versions of the instrument to improve internal consistency and/or construct validity [4, 16]. In the United States, the study used exploratory factor analysis on each scale and an improved model fit was obtained with a shortened version with 49 items [16]. Most alpha coefficients were over 0.70, except for the scale empathy/tolerance in the ‘individual’ domain. The total score of the 88-item ARQ was highly correlated to the new ARQ with 49 items [16]. In the same way, the Indian version with five domains and 35 items showed an acceptable model fit [4].

The B-ARQ-SV had a satisfactory factor structure once we grouped the connectedness and availability scales on the ‘peers’ domain. It is possible that the high correlation between these scales in the Brazilian version may indicate that emotions regarding their peers are closely related, reflecting the culture of the country. However, our choice to join the aforementioned scales was also due to the similarity between them. Future studies might benefit from a more in-depth analysis of this domain in the population and the use of different methods such as qualitative study designs. Moreover, in the CFA, some items were not significant (p>0.05); however, the study assessed resiliency in a sample of adolescents from Southern Brazil, and the results might be different in the assessment of other populations. Our choice to include these items in the final version of the instrument sought to ensure the use of the instrument as originally designed, enabling comparison from data obtained from populations across different countries.

Absolute fit indexes such as SRMR and RMSEA assess if the data in the sample is well reproduced according to a previous model [26]. The fit indexes that assessed the model fit of the short Brazilian version presented satisfactory scores, as proposed by the American 49-item version [16]. Moreover, using the shortened version of questionnaires might have its benefits. For instance, in long instruments with many items, the possibility of a response fatigue bias is a matter of concern [28], which echoes the suggestions of volunteers in our study. In the pretest, they reported the 88-item ARQ as a rather long instrument and suggested a shortened version might be more adequate.

In the B-ARQ-SV the frequency of respondents who scored the highest mark (ceiling effect) was over 15% in only one domain (scale availability, ’family’ domain); and there was no floor effect—the frequency of individuals who scored the lowest mark was below 15%. Such findings may be interpreted as part of content validity and a positive assessment of instrument quality, as it is able to correctly distinguish people with different levels of the spectrum [27]. In this way, should researchers and clinicians look for an instrument to assess resilience among adolescents, this is one relevant measure of quality that should be taken into consideration.

The findings presented here should be interpreted considering certain limitations. The study was carried out in a sample of adolescents from a city in Southern Brazil, a culturally diverse country. Hence it is not possible to guarantee that study results are representative of youth patients from different Brazilian regions. Also, factor loadings of the B-ARQ-SV was below 0.40 in several items; however, results might be different in future studies that use the Brazilian version of the instrument in a representative sample of Brazilian adolescents. Future studies using the B-ARQ-SV in a wider sample of adolescents from different regions and backgrounds are encouraged, as well as studies that compare the data obtained with the B-ARQ-SV and other instruments measuring resilience or even similar constructs such as anxiety or quality of life. In addition, further analysis of the instrument such as item response theory, and application to new populations is needed.

## Conclusion

The 49-item Brazilian short version of the ARQ (B-ARQ-SV) based on first-order model with 11 scales is a valid (in terms of content and structural validity) and reliable (in terms of internal consistency) measurement instrument to assess resilience in Brazilian adolescents.

## Supporting information

S1 File Dataset Brazilian version of the Adolescent Resilience Questionnaire (B-ARQ). (XLSX)
